# *Forsythia suspensa* (Thunb.) Vahl extract ameliorates ulcerative colitis via inhibiting NLRP3 inflammasome activation through the TLR4/MyD88/NF‐κB pathway

**DOI:** 10.1002/iid3.1069

**Published:** 2023-11-07

**Authors:** Xiao Tong, Li Chen, Shijun He, Shuangshuang Liu, Jiaying Yao, Zhenguang Shao, Yang Ye, Sheng Yao, Zemin Lin, Jianping Zuo

**Affiliations:** ^1^ University of Chinese Academy of Sciences Beijing China; ^2^ State Key Laboratory of Drug Research Chinese Academy of Sciences Shanghai China; ^3^ Innovation Research Institute of Traditional Chinese Medicine Shanghai University of Traditional Chinese Medicine Shanghai China; ^4^ College of Pharmacy Jiangxi University of Traditional Chinese Medicine Nanchang China; ^5^ State Key Laboratory of Drug Research & Natural Products Research Center Chinese Academy of Sciences Shanghai China; ^6^ School of Life Science and Technology ShanghaiTech University Shanghai China; ^7^ Zhongshan Institute for Drug Discovery Chinese Academy of Sciences Zhongshan China; ^8^ Laboratory of Immunology and Virology Shanghai University of Traditional Chinese Medicine Shanghai China

**Keywords:** macrophages, NLRP3 inflammasome, phillygenin, ulcerative colitis

## Abstract

**Background:**

Ulcerative colitis (UC), a chronic inflammatory disease, is caused by abnormal immune system reactions resulting in inflammation and ulcers in the large intestine. Phillygenin (PHI) is a natural compound found in *Forsythia suspensa* (Thunb.) Vahl, which is known for its antipyretic, anti‐inflammatory, antiobesity, and other biological activities. However, the therapeutic role and molecular mechanisms of PHI on UC are still insufficiently researched.

**Methods:**

In this study, dextran sulfate sodium (DSS) and 2.5% 2,4,6‐trinitro‐Benzenesulfonic acid (TNBS)‐induced acute UC were used to investigate the therapeutic effects of PHI. We evaluated the effects of PHI on disease activity index (DAI), body weight, mortality, intestinal mucosal barrier, cytokine secretion, and macrophage infiltration into colon tissue using various techniques such as flow cytometry, immunofluorescence, enzyme‐linked immunosorbent assay (ELISA), RT‐qPCR, and Western blot analysis.

**Results:**

Our findings revealed that PHI has therapeutic properties in UC treatment. PHI was able to maintain body weight, reduce DAI and mortality, restore the intestinal mucosal barrier, and inhibit cytokine secretion. Flow cytometry assay and immunofluorescence indicated that PHI reduces macrophage infiltration into colon tissue. Mechanistically, PHI may exert anti‐inflammatory effects by downregulating the TLR4/MyD88/NF‐κB pathway and inhibiting the activation of NLRP3 inflammasome.

**Conclusion:**

In conclusion, PHI possesses significant anti‐inflammatory properties and is expected to be a potential drug for UC treatment. Our study delves into the underlying mechanisms of PHI therapy and highlights the potential for further research in developing PHI‐based treatments for UC.

## Graphical abstract

*Forsythia suspensa* (Thunb.) Vahl extract (PHI) ameliorated both DSS‐ and TNBS‐induced inflammatory bowel disease (IBD) in mice PHI reduced the proportion of inflammatory cells in mesenteric lymph node and peripheral blood mononuclear cells of IBD mice, especially reducing macrophage infiltration into colon PHI inhibited NLRP3 inflammasome activation in IBD mice by downregulating the TLR4/MyD88/NF‐κB signaling pathway, providing a basis for traditional Chinese medicine application in treating this disease.

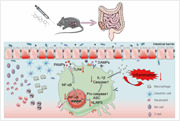

**Abbreviations**
ASC: apoptosis‐associated speck‐like protein containing CARD
ATP: adenosine triphosphate
BMDM: bone marrow‐derived macrophage
ConA: concanavalin A
DAI: disease activity index
DAPI: 4′,6‐diamidino‐2‐phenylindole
DSS: dextran sulfate sodium
H&E: hematoxylin and eosin
IBD: inflammatory bowel disease
LPS: lipopolysaccharide
M‐CSF: mouse colony‐stimulating factor
MLN: mesenteric lymph nodes
NF‐κB: nuclear factor‐κB
NLRP3: NOD‐like receptor family pyrin domain containing 3
PAMP: pathogen‐associated molecular pattern
PAS: periodic acid‐Schiff stain
PBMC: peripheral blood mononuclear cell
PHI: phillygenin
TLR: Toll‐like receptor
TNBS: 2,4,6‐trinitro‐Benzenesulfonic acid
UC: ulcerative colitis

## 1 BACKGROUND

Ulcerative colitis (UC) is an immune‐mediated inflammatory disease that occurs in the colon and rectum. It is characterized by diffuse, superficial, and localized inflammation of the mucosa.^1^, ^2^ Although the causative factors of UC are not fully understood, multiple causes have been identified, including changes in the intestinal flora, genetic susceptibility, immune response disorders, and environmental factors. Clinical treatment usually involves thiopurines, corticosteroids, 5‐aminosalicylic acid, and biological agents. However, these treatments can cause specific side effects, including diarrhea, complicated myocarditis, and hemolytic anemia.^3^

Macrophages, which is considered as central mediators of intestinal immune homeostasis and inflammation, exert pathological influences in both UC and Crohn's.^4^ In response to pathogen‐associated molecular patterns (PAMPs), Toll‐like receptor (TLR) is activated, which in turn activates macrophages leading to excessive inflammation and tissue damage, causing colitis.^5^ Moreover, innate immune signaling mediated macrophages activation act as a primary response to promote the transcription of NOD‐like receptor family pyrin domain containing 3 (NLRP3) through nuclear factor‐κB (NF‐κB) activation.^6^

The NLRP3 inflammasome is a cytosolic protein complex, composed of NLRP3, apoptosis‐associated speck‐like protein containing CARD (ASC), and pro‐Caspase1. This complex is present in various immune cells and is crucial for maintaining gut homeostasis.^7^ TLRs activate the NLRP3 inflammasome with the help of the adaptor protein (myeloid differentiation factor 88, MyD88), subsequently leads to the phosphorylation of the NF‐κB and triggers downstream inflammation. The NLRP3 inflammasome identifies a range of signals such as stress, foreign microorganisms, and endogenous danger signals, producing IL‐18 and interleukin‐1β (IL‐1β), thus promoting inflammation.^8^, ^9^ IL‐1β is derived primarily from macrophages in the laminae propria and performs a variety of functions in the colon, including guiding neutrophils to infected or damaged sites, enhancing T cell proliferation, promoting phagocytosis to destroy bacteria, and activating additional pathways to upregulate cytokines, among others.^8^, ^10^

*Forsythia suspensa* (Thunb.) Vahl is an ornamental shrub and its fruits were used as a well‐known Chinese herbal medicine with detoxifying and heat‐clearing properties. Over the past few decades, several monomeric compounds with antioxidant, anti‐inflammatory, antiviral, neuroprotective, and antibacterial effects have been identified from *Forsythia suspensa*.^11^ One of these compounds, phillygenin (PHI), is a lignan known to inhibit inflammation, ameliorate liver fibrosis, inhibit epithelial‐mesenchymal transition, reduce nonalcoholic fatty liver disease, and regulate proliferation and apoptosis.^12^, ^13^, ^14^, ^15^ Previous studies have found that *Forsythia suspensa* Fructus has the potential for treatment of intestinal inflammation.^16^ Recently, Feng et al. have reported that PHI alleviated dextran sulfate sodium (DSS)‐induced UC through the Nrf2‐NLRP3 pathway and improved metabolic dysfunction.^17^, ^18^, ^19^ In spite of this, the mechanism of PHI in the treatment of UC has not been thoroughly studied. In the current study, we performed a more detailed study on the pharmacological impact and mechanism of PHI on colitis.

## 2 METHODS

### 2.1 Drugs

Preparation of PHI via phillyrin hydrolysis: Naturally occurring PHI (from an in‐house natural product library, >97% purity), Synthetic phillyCgenin (>98% purity) for in vivo study from phillyrin hydrolysis (Supporting Information: Figures S1–S5). The extraction of PHI was extracted, identified, and characterized. Briefly, Phillyrin (3.0 g, 5.62 mmol) was dissolved in 120 mL of methanol, to this solution was added 2 M HCl (24 mL, 12 mmol, 8.6 eq.). The reaction was refluxed at 65°C overnight, then cooled to room temperature and adjust pH to 5~6. The resulting mixture was evaporated under reduced pressure and then was extracted three times with EtOAc (100 mL) three times. The organic phase was washed twice with brine (100 mL), then dried and evaporated. The residue was eluted with dichloromethane/methanol (20/1) to yield PHI a silica gel column.

### 2.2 Animals

BALB/c mice (female, 6–8 weeks), C57BL/6 mice (male and female, 8 weeks) were purchased from Beijing Huafukang Biotechnology Co., Ltd. The animals were kept in a specific pathogen‐free environment with controlled conditions, including a 12‐h light/dark cycle, 22 ± 1°C temperature, and 55 ± 5% relative humidity. All experiments were performed under the guide of the National Institutes of Health Guide for Care and Use of Laboratory Animals, and were approved by the Bioethics Committee of the Shanghai Institute of Materia Medica.

### 2.3 Drug treatment and assessment of DSS‐induced colitis

C57BL/6 mice were randomly divided into four group (a normal control group, a vehicle control group, 60 mg/kg PHI treatment group and 20 mg/kg PHI treatment group). The normal group received sterile water throughout the experiment, while the other groups received 2% DSS (MP Biomedicals) in the first 7 days and regular drinking water in the last 3 days remission. During the experiment, the body‐weight loss ratio, stool consistency and rectal bleeding were assessed daily according to the described criterion (see Table 1). The total score of these parameters represents the disease activity index (DAI). On Day 10, mice were anesthetized, and the peripheral blood, colon, and mesenteric lymph nodes (MLNs) were collected for subsequent analysis.

**Table 1:** Criteria for weight loss, stool consistency and fecal blood.

Score	Weight loss (%)	Stool consistency	Blood
0	0	Normal	None
1	1–5	Slightly soft	Weakly occult blood
2	6–10	Soft	Positive hemoccult
3	11–20	Slightly diarrhea	Visible blood
4	>20	Watery diarrhea	Bloody stool

### 2.4 Drug treatment and assessment of TNBS‐induced colitis

C57BL/6 mice were randomly divided into four groups (Normal group, Vehicle group, 60 mg/kg PHI treatment group and 20 mg/kg PHI treatment group). The last three group of mice received 2.5% 2,4,6‐trinitro‐Benzenesulfonic acid (TNBS; Sigma‐Aldrich) by clysma on Day 0 of the experiment. The drug treatment group received 40 mg/kg PHI orally. The body‐weight change and survival rate were monitored daily. At the end of the experiment, mice were anesthetized, and the peripheral blood, colon, and MLNs were collected for subsequent analysis.

### 2.5 Histological analysis

10% formalin‐fixed and paraffin‐embedded colons tissue specimens were stained with Hematoxylin and eosin (H&E) to observe the colon tissue pathology under an optical microscope. The scoring criteria consisted of our previously published system.^20^, ^21^ Additionally, periodic acid‐Schiff stain (PAS) and Alcian blue staining techniques were used to examine the epithelial goblet cells, characterizing neutral mucin and acidic mucins, respectively. The Leica DM6B laser microdissection system was utilized to obtain images.

### 2.6 FITC‐dextran intestinal permeability assay

After overnight fasted, mice were orally administered 600 mg/kg of FITC‐dextran (Sigma‐Aldrich). After 4 h, their serum was collected and the fluorescence intensity was measured (Ex: 480 nm, Em: 520 nm).

### 2.7 Flow cytometry assay

Single cell suspension was prepared by grinding MLNs and peripheral blood mononuclear cells (PBMCs) suspension was also prepared. Then the single cells were blocked with purified anti‐mouse CD16/CD32 mAb for blocking and separately dyed flow cytometry antibodies: BUV395‐conjugated anti‐CD11b mAb, FITC‐conjugated anti‐CD11c mAb, PE‐conjugated anti‐Ly6G mAb, BV510‐conjugated anti‐F4/80 mAb, BUV395‐conjugated anti‐CD4 mAb, Percp‐cy5.5‐conjugated anti‐CD3 mAb and PE‐conjugated anti‐CD69 mAb (BD Bioscience). After incubating, the cells were washed once with phosphate buffer saline and analyzed using Fortessa Flow Cytometer (BD Bioscience). The data were analyzed with FlowJo software.

### 2.8 Cell culture and treatment

Splenocytes extracted from BALB/c mice were cultured in triplicate for 48 h and treated with different concentrations of PHI. Meanwhile, mitogen concanavalin A (Con A, 5 μg/mL) and lipopolysaccharide (LPS, 10 μg/mL) were used to induce lymphocytes abnormal proliferation. The cells were pulsed with 0.5 μCi/well of [^3^H] thymidine at the last 8 h and the incorporated radioactivity was counted using a Beta Scintillation Counter (MD SpectraMAX190).

Bone marrow‐derived macrophages (BMDMs) were separated from the femur and tibia bones of BALB/c mice and then cultured for 7 days in RPMI‐1640 medium containing 10% fetal bovine serum and 10 ng/mL of Colony‐stimulating factor (M‐CSF; Peprotech). Differentiation of THP‐1 cells was induced by 5 ng/mL PMA (phorbol 12‐myristate 13‐acetate) for 48 h. The differentiated BMDMs or THP‐1 were treated with 1 μg/mL LPS in the absence or presence of PHI.

### 2.9 Immunofluorescence

For colon, the dehydrated and rehydrated tissues were blocked with 5% bovine serum albumin and then stained with anti‐F4/80 and counterstained with 4′,6‐diamidino‐2‐phenylindole (DAPI). The F4/80 signals were detected by Alexa Fluor 647 conjugate goat anti‐rabbit IgG. For BMDMs, cells are fixed by 4% paraformaldehyde, permeated by 1% Triton X‐100, and blocked by immunostaining blocking solution. Anti‐NLRP3 is used to incubate cells overnight, then detected with Alexa Fluro 488 conjugate goat anti‐rabbit IgG staining. The cell nucleus is then stained by DAPI. Captured images are analyzed using the Leica TCS SPS microscope.

### 2.10 RNA extraction and real‐time quantitative polymerase chain reaction (RT‐qPCR)

RNA lysate RZ is used to homogenize mice colons, followed by RNA extraction using the RNAsimple total RNA kit (Tiangen Biotech). Reverse transcription is carried out with HifairTM Ⅱ 1st strand cDNA synthesis supermix for qPCR (Yeasen), and real‐time quantitative PCR is performed with SYBR Green Realtime PCR Master Mix (Yeasen). PCR amplification is performed with the primers listed in Table 2.

**Table 2:** Sequences of primers.

Genes	Forward (5′‐3′)	Reveres (5′‐3′)
Mouse β‐actin	GGCTGTATTCCCCTCCATCG	CCAGTTGGTAACAATGCCATGT
Mouse NLRP3	ATTACCCGCCCGAGAAAGG	TCGCAGCAAAGATCCACACAG
Mouse ASC	AGACATGGGCTTACAGGAGCTG	CCACAAAGTGTCCTGTTCTGGC
Mouse Caspase 1	ACAAGGCACGGGACCTATG	TCCCAGTCAGTCCTGGAAATG
Mouse TNF‐α	ATGTCTCAGCCTCTTCTCATTC	GCTTGTCACTCGAATTTTGAGA
Mouse IL‐1β	GCAACTGTTCCTGAACTCAACT	ATCTTTTGGGGTCCGTCAACT
Mouse IL‐6	CCAAGAGGTGAGTGCTTCCC	CTGTTGTTCAGACTCTCTCCCT
Mouse TLR4	ATGGCATGGCTTACACCACC	GAGGCCAATTTTGTCTCCACA
Human β‐actin	TTGTTACAGGAAGTCCCTTGCC	ATGCTATCACCTCCCCTGTGTG
Human TNF‐α	CCTCTCTCTAATCAGCCCTCTG	GAGGACCTGGGAGTAGATGAG
Human IL‐1β	AGCTACGAATCTCCGACCAC	CGTTATCCCATGTGTCGAAGAA
Human IL‐6	ACTCACCTCTTCAGAACGAATTG	CCATCTTTGGAAGGTTCAGGTTG

### 2.11 Western blot analysis

The cultured cells and tissues were lysed with sodium dodecyl sulfate (SDS) buffer containing protease inhibitor cocktail (Roche Life Science), and the protein concentration was detected with BCA protein assay kit (Thermo Fisher Scientific). After adjusting to the same concentration, the protein were separated by SDS‐PAGE and blotted with antibodies against Occludin, TLR4 (Abcam), E‐cadherin, NF‐κB, p‐NF‐κB, Cleaved Caspase‐1, MyD88, NLRP3, ASC (Cell Signaling Technology) and GAPDH (KangChen). Proteins were visualized using HRP‐conjugated anti‐rabbit or anti‐mouse IgG and the densities of the bands were quantified with ImageJ 1.42.

### 2.12 Statistical analysis

Statistical differences were detected by GraphPad Prism 8.3. All data were analyzed by one‐way analysis of variance with Dunnet's multiple comparisons tests or two‐tailed Student's *t* test. *p* < .05 were considered significant.

## 3 RESULTS

### 3.1 PHI exhibited immunosuppressive activity in vitro

**Figure 1:**
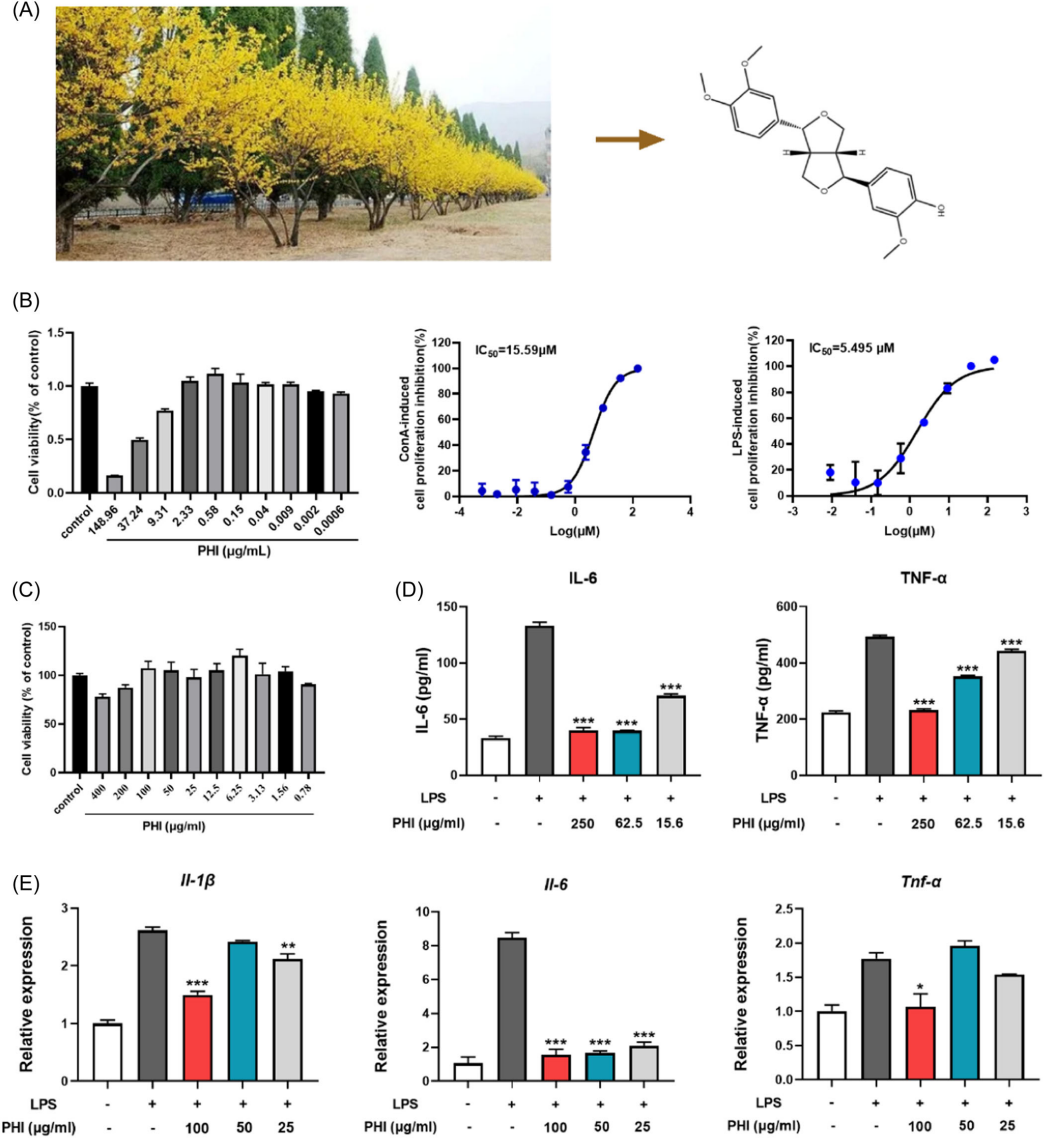
PHI exhibited favorable immunosuppressive activity in vitro. (A) Chemical structure of PHI. (B) Cell viability of splenic lymphocytes and IC_50_ on ConA‐ or LPS‐induced lymphocyte proliferation by PHI treatment. (C) Cell viability of THP‐1 cells by PHI treatment. (D) Cytokine secretion levels in LPS‐induced THP‐1 culture. (E) The gene expression level of cytokines in LPS‐induced THP‐1 cells. Compared with the LPS ‐stimulated group. ConA, concanavalin A; LPS, lipopolysaccharide; PHI, phillygenin. *p＜0.05, **p＜0.01, ***p＜0.001, *n* = 3–4.

The extraction of PHI was extracted, identified, and characterized (Figure 1A, Supporting Information: Figure S1–S5). The immunosuppressive activities of PHI on murine splenocytes and monocyte‐derived macrophages THP‐1 were evaluated. As shown in Figure 1B,C, the CC_50_ of PHI on murine splenocytes was found to be 35.43 μg/mL, and PHI exerted ideal immunosuppressive effects on lymphocyte proliferation induced by ConA and LPS, with IC_50_ values of 4.357 and 1.539 μg/mL, respectively. Meanwhile, PHI effectively inhibited the secretion of proinflammatory cytokines induced by LPS (Figure 1D,E). Taken together, these results indicate that PHI is a promising candidate for immunosuppressive therapy in vitro.

### 3.2 PHI ameliorated DSS‐induced murine acute UC

**Figure 2:**
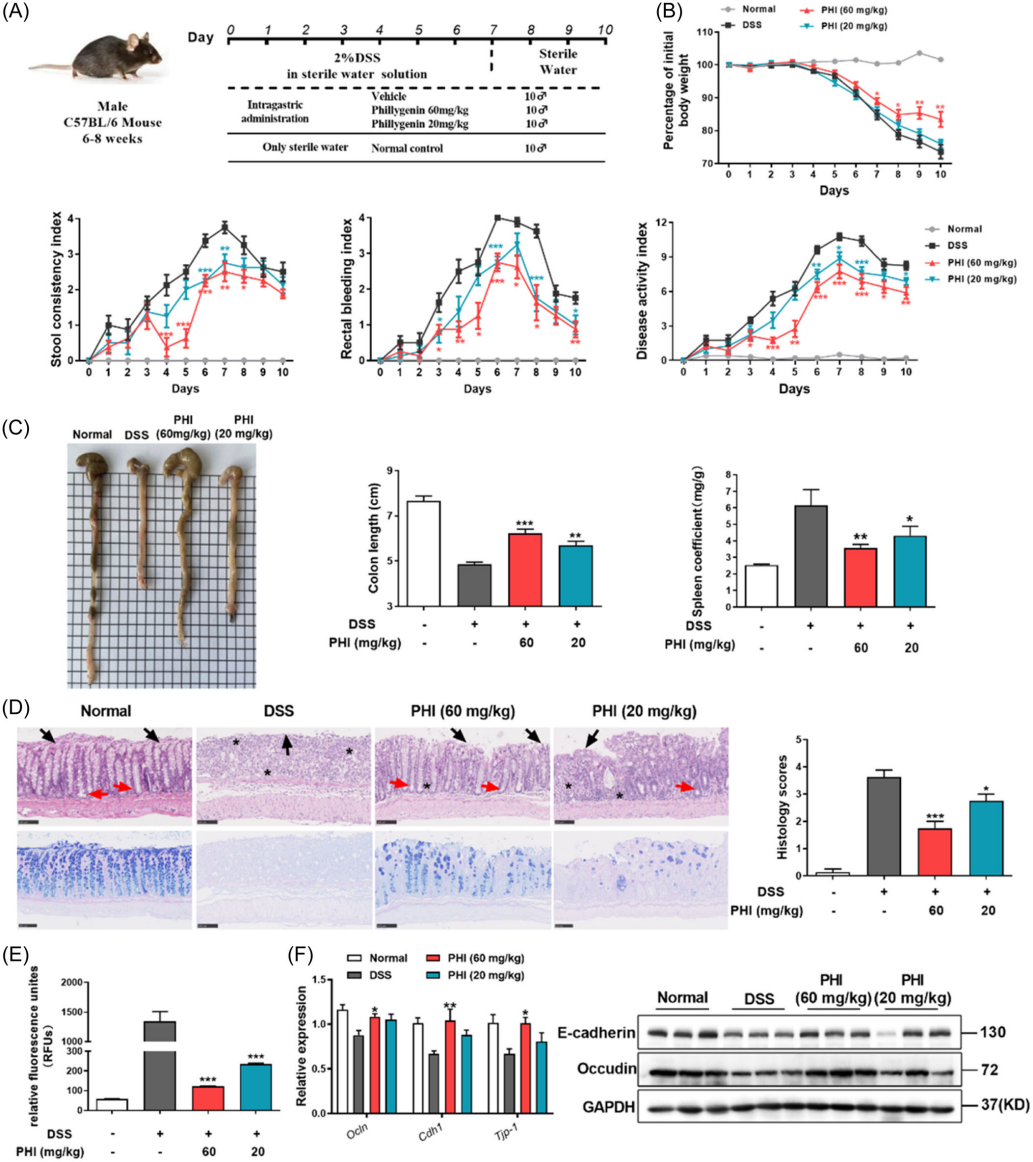
PHI treatment ameliorated DSS‐induced murine acute UC. (A) DSS‐induced murine model. (B) Body weight change, diarrhea, stool bleeding, and DAI. (C) Representative images of colon morphology, quantitative colon length, and the spleen coefficient. (D) Representative images of H&E, PAS‐Alcian blue staining of colon sections, and histological scores, bar = 100 μm. Asterisks referred to inflammatory cell infiltration. Black arrows referred to epithelial cells while red arrows referred to crypts. (E) Fluorescence intensity of FITC‐dextran in serum. (F) The mRNA and protein levels of tight junction proteins in the intestine. Compared with the vehicle (DSS) group. DAI, disease activity index; DSS, dextran sulfate sodium; H&E, hematoxylin and eosin; PAS, periodic acid‐Schiff stain; PHI, phillygenin; UC, ulcerative colitis. *p＜0.05, **p＜0.01, ***p＜0.001, *n* = 6–8.

We used DSS‐induced colitis to investigate the therapeutic effects of PHI. Two percent DSS in drinking water to establish the UC model for 7 consecutive days, another 3 days of normal drinking water in remission. Treating with different doses of PHI (60 and 20 mg/kg) throughout the whole experiment (Figure 2A).

Using DAI as a measure of UC severity, the mice in DSS‐induced colitis experienced significant weight loss, severe diarrhea, and bloody stool after chemical induction. Treatment with PHI resulted in a reversal of these symptoms, as well as a restoration of immune hyperfunction‐induced splenomegaly and colon shortening (Figure 2B,C). In addition, the H&E staining showed that the colonic structure of DSS‐induced UC mice was lost, crypts were destroyed and inflammatory cells infiltrated seriously. PHI treatment improved these phenomenons. Besides, PHI exerted positive effects on maintaining the intestinal mucosal barrier by reducing goblet cell loss, mucous thinning, and intestinal permeability as showing by PAS‐Alcian blue staining (Figure 2D). In addition, the expression of tight junction proteins in the colon was restored, further demonstrating PHI's beneficial effect on intestinal barrier function (Figure 2E,F).

### 3.3 PHI ameliorated TNBS‐induced murine UC

**Figure 3:**
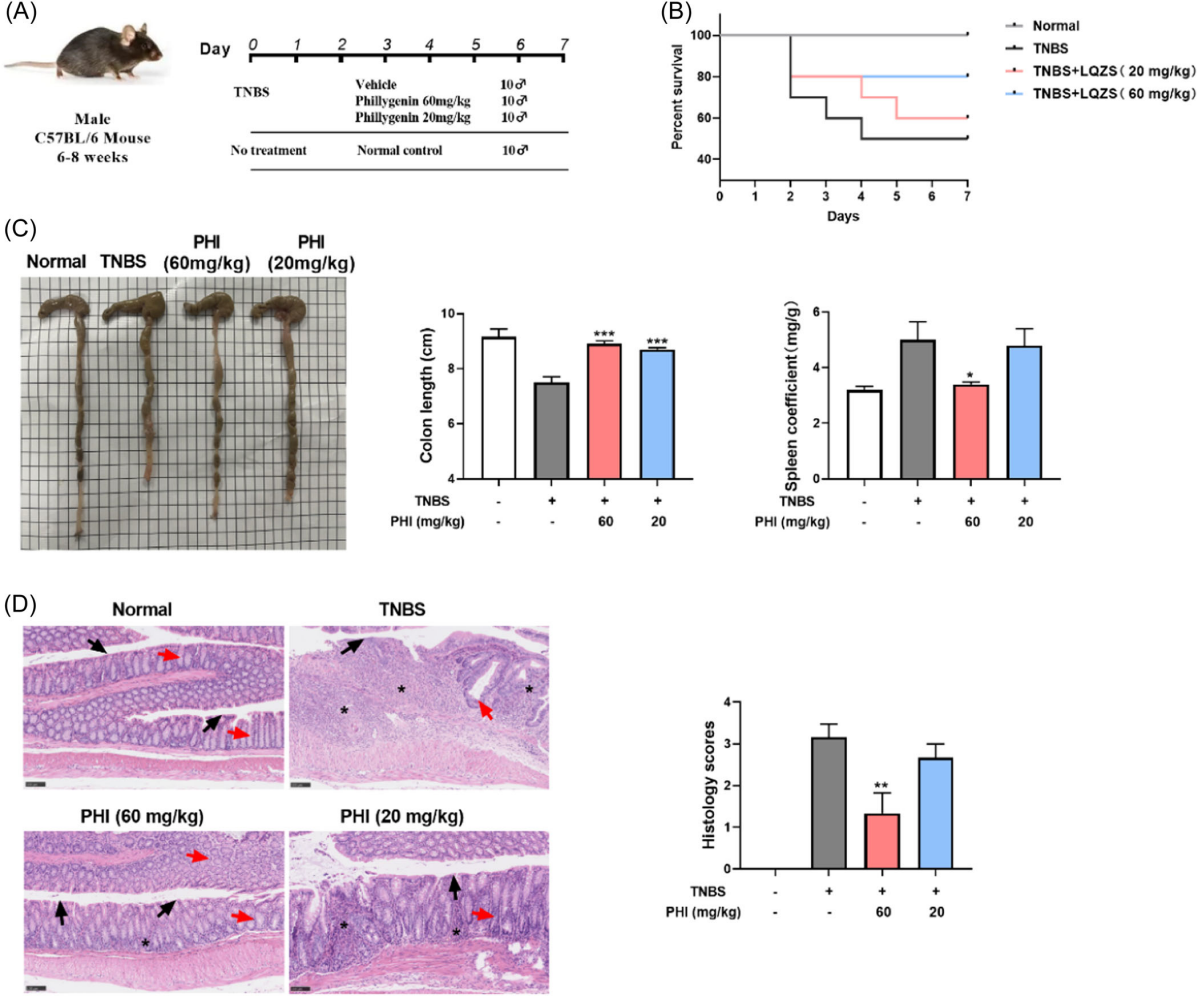
PHI ameliorated TNBS‐induced murine acute UC. (A) TNBS‐induced murine model. (B) Survival rate. (C) Representative images of colon morphology, quantitative colon length, and the spleen coefficient. (D) Representative images of H&E staining and histological scores, bar = 100 μm. Asterisks referred to inflammatory cell infiltration. Black arrows referred to epithelial cells while red arrows referred to crypts. Compared with the model control group. H&E, hematoxylin and eosin; PHI, phillygenin; TNBS, 2,4,6‐trinitro‐Benzenesulfonic acid; UC, ulcerative colitis. *p＜0.05, **p＜0.01, ***p＜0.001, *n* = 5–10.

To better explore the effect of PHI on inflammatory bowel disease (IBD), we also used 2.5% TNBS to induce gut inflammation. Mice were treated with 60 and 20 mg/kg PHI from Day 0 to the endpoint (Figure 3A). Similarly, PHI significantly increased the survival rate, restored colon length, and maintained spleen coefficient of TNBS‐induced UC mice (Figure 3B,C). Compared with TNBS group, the crypts deficiency, mildly intestinal edema and inflammatory cell infiltration in PHI‐treated group was significantly improved (Figure 3D). Overall, the study provides evidence that PHI has a favorable pharmaceutical effect in different chemically induced UC models and is expected to be a UC therapeutic agent.

### 3.4 PHI reduced the infiltration of inflammatory cells

**Figure 4:**
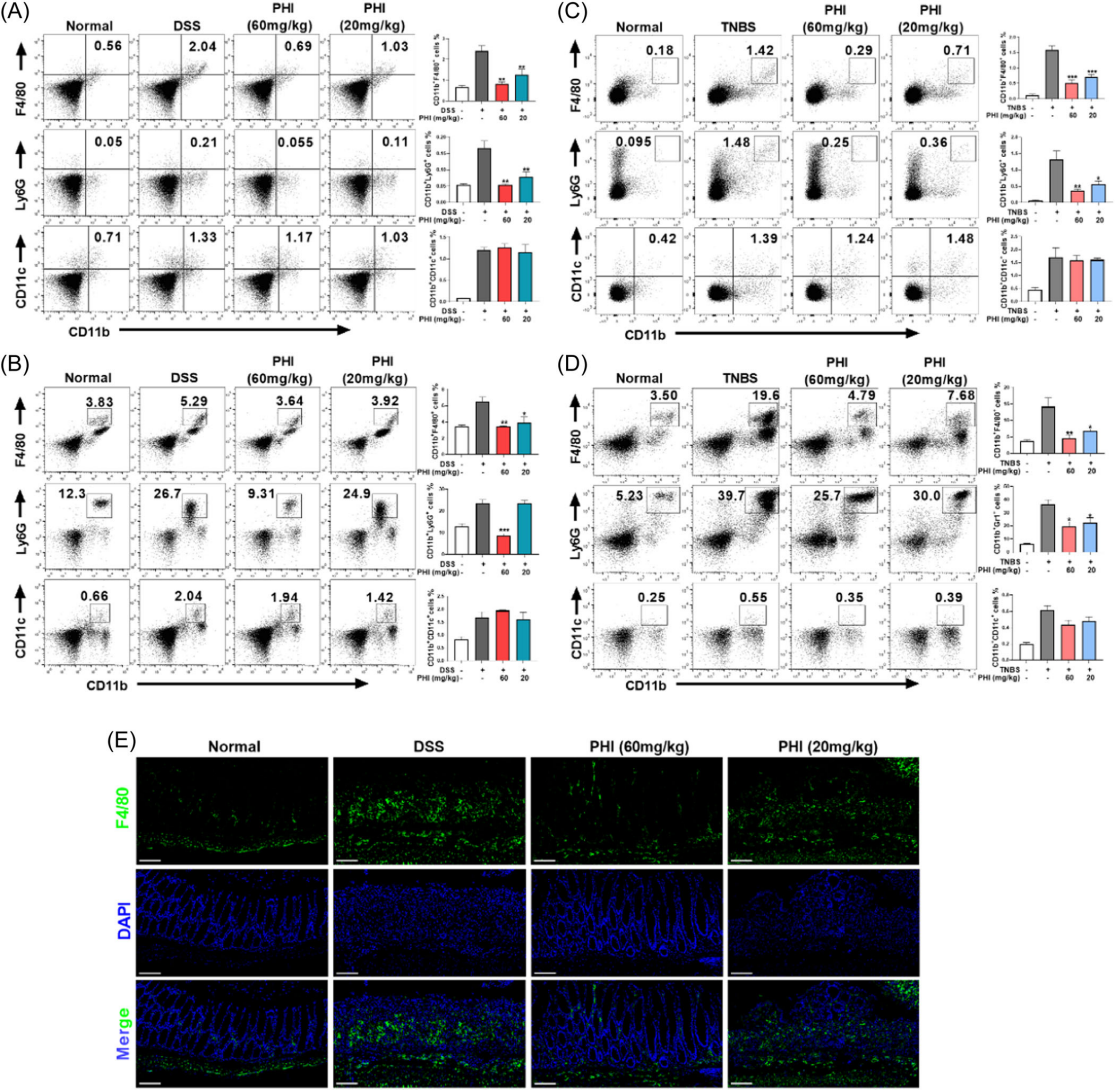
PHI inhibited inflammatory cells in MLN and PBMC of DSS and TNBS‐induced UC. (A and B) Flow cytometry and quantitative analysis of MLNs and PBMCs in the DSS‐induced UC model, including macrophages (CD11b^+^F4/80^+^), neutrophils (CD11b^+^Ly6G^+^), and dendritic cells (CD11b^+^CD11c^+^). (C and D) Flow cytometry and quantitative analysis of MLNs and PBMCs in TNBS‐induced UC model. (E) Representative images of immunofluorescence staining of F4/80, bar = 100 μm. Compared with the DSS group. DSS, dextran sulfate sodium; MLN, mesenteric lymph nodes; PHI, phillygenin; PBMC, peripheral blood mononuclear cell; TNBS, 2,4,6‐trinitro‐Benzenesulfonic acid; UC, ulcerative colitis. *p＜0.05, **p＜0.01, ***p＜0.001, *n* = 3–4.

Neutrophils and macrophages infiltrations are recognized as evaluative parameters for the severity degree of colitis. The population of macrophages (CD11b^+^F4/80^+^), neutrophils (CD11b^+^Ly6G^+^) and dendritic cells (CD11b^+^CD11c^+^) were significantly increased in both MLNs and PBMCs of DSS‐induced UC mice. Both high and low doses of PHI were found to reduce the population of macrophages in MLNs and PBMCs, but only high doses of PHI having an inhibitory effect on neutrophils of PBMCs (Figure 4A,B). In TNBS‐induced mice, the population of macrophages decreased, and PHI reduced the level, which was consistent with the findings in DSS‐induced mice (Figure 4C,D). Immunofluorescent staining with F4/80 showed severe infiltrations of macrophages in DSS‐induced mice colon, which were reduced by PHI (Figure 4E), suggesting that future research may focus on macrophages' role in the mechanism of action of PHI.

### 3.5 PHI improved UC by inhibiting NLRP3 inflammasome activation through TLR4/MyD88/NF‐κB pathway

**Figure 5:**
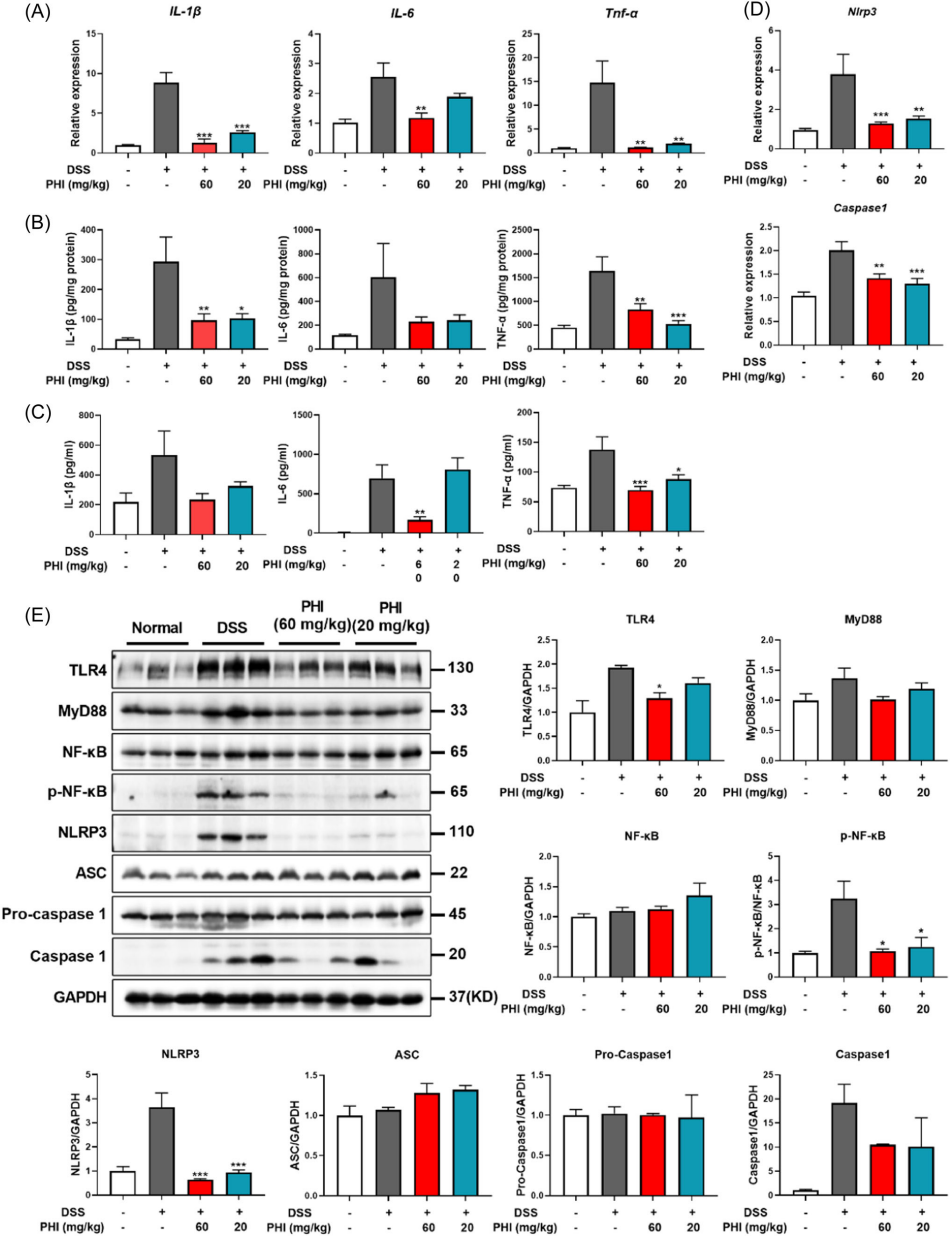
Phillygenin (PHI) ameliorated inflammation via the NLRP3 inflammasome pathway. (A) The gene expression level of cytokines in colonic homogenates. (B and C) The protein levels of cytokines in colonic homogenates and serum. (D) The gene expression level of *Nlrp3* and *Caspase 1*. (E) The protein expression of NLRP3 inflammasome and its upstream pathway. Compared with the model control group. *p＜0.05, **p＜0.01, ***p＜0.001, *n* = 3–4.

Proinflammatory mediators such as cytokines IL‐1β, IL‐6, and tumor necrosis factor α (TNF‐α) play key roles in UC, and the secretion level of these cytokines in serum or colon was found to rise sharply in the presence of UC. After PHI treatment, these inflammatory factors were significantly inhibited in the colon and serum (Figure 5A–C).

NLRP3 inflammasome was reported to be an important protein complex for IL‐1β production. Given the remarkable effect of PHI on IL‐1β, we detected the expression level of NLRP3 inflammasome relative proteins. As shown in Figure 5D,E, the expression of both NLRP3 and Caspase1 was increased dramatically with DSS induction, which was restrained in PHI‐treated group.

### 3.6 PHI improved LPS and adenosine triphosphate (ATP)‐induced BMDM inflammation by inhibiting NLRP3 inflammasome activation

**Figure 6:**
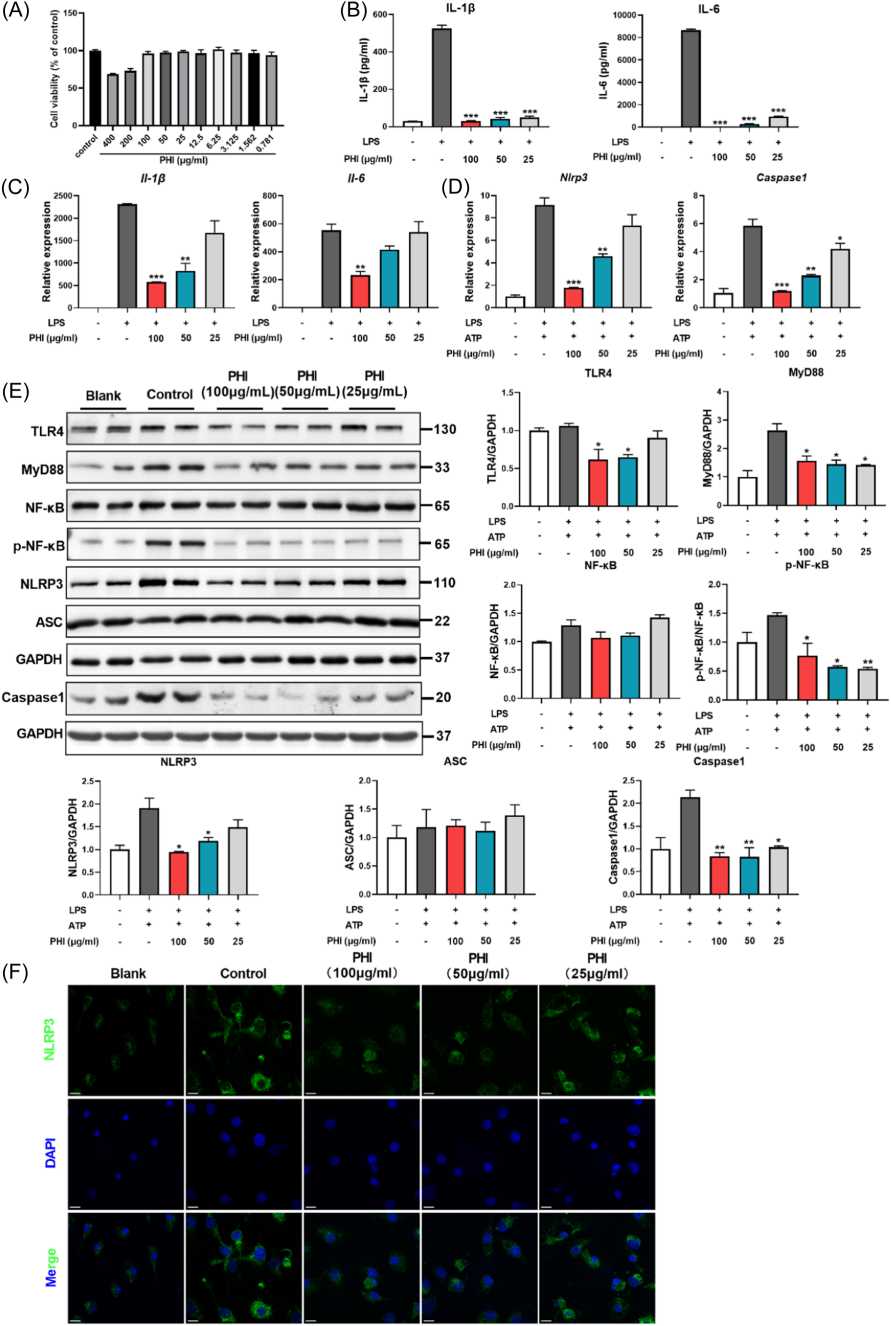
PHI inhibited the inflammation of BMDMs by inhibiting the NLRP3 inflammasome pathway. (A) Cell viability of BMDMs by PHI treatment. (B and C) The protein and gene expression levels in LPS‐induced BMDMs. (D) The gene expression level of *Nlrp3* and *Caspase 1*. (E) The protein expression of inflammasome and its upstream pathway. (F) Representative images of immunofluorescence staining of NLRP3, bar = 10 μm. Compared with the LPS or LPS&ATP‐stimulated group. BMDM, bone marrow‐derived macrophage; LPS, lipopolysaccharide; PHI, phillygenin. *p＜0.05, **p＜0.01, ***p＜0.001, *n* = 3–4.

Macrophages are known to play a critical role in the progression of inflammation and the eventual remission of disease during intestinal mucosal repair. To investigate the efficacy of PHI in vitro, BMDMs were utilized in an in vitro experiment due to their significant impact on macrophages compared with other myeloid cells. In the initial phase of the experiment, the impact of PHI on cell viability was examined, and three safe concentrations were selected (100, 50, and 25 μg/mL) for subsequent research (Figure 6A). BMDM was then stimulated with LPS, a typical TLR4 agonist, to induce inflammatory cytokine production. PHI displayed potent anti‐inflammatory properties on BMDM, which was reflected in a reduction expression of IL‐1β and IL‐6 under LPS induction (Figure 6B,C).

**Figure 7:**
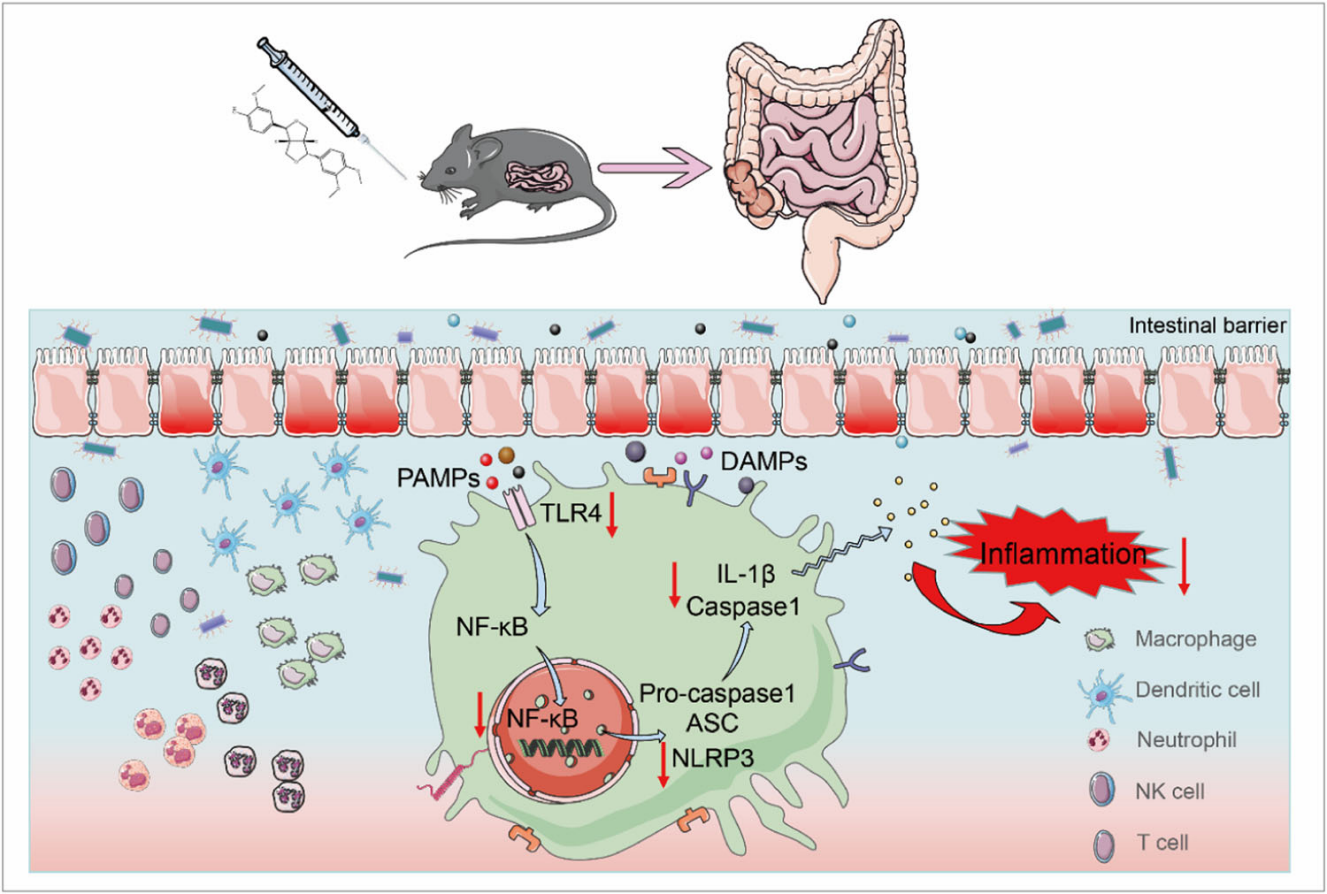
Graphical summary of amelioration of ulcerative colitis by PHI via inhibiting NLRP3 inflammasome activation through the TLR4/MyD88/NF‐κB pathway.

Based on these findings, we tested the effect of PHI on NLRP3 inflammasome in vitro. The NLRP3 inflammasome was activated by LPS and NLRP3 agonist ATP. As with the results in UC mice colon, PHI inhibited the expression of NLRP3 and Caspase1, especially NLRP3, which was also demonstrated by using cellular immunofluorescence (Figure 6D–F). The influence mechanism was also proven, as it inhibited the upstream pathway as a depressant. By combining the results in both in vivo and in vitro experiments, the conclusion was that PHI had a positive effect on UC, and it inhibited inflammation by preventing NLRP3 inflammasome activation through the TLR4/MyD88/NF‐κB pathway (Figure 7).

## 4 DISCUSSION

UC is an inflammatory disease characterized by inflammation of the colon mucosa, which affects the rectum and extends proximally.^22^ The incidence of UC has been increasing every year due to the industrialization of various countries and the westernization of people's eating habits.^23^ Although there have been therapeutic advancements in UC, there is still a treatment gap from people's ideal state.^24^ Consequently, recent research has increasingly shown the potential use of active monomers in functional food or traditional Chinese medicine to treat colitis.^25^ *Forsythia suspen*sa, a deciduous shrub with bitter and cold characteristics that align with anti‐inflammatory Chinese medicine, has been used historically for fever, inflammation, gonorrhea, and erysipelas. Through exploring the immunosuppressive activity of *Forsythia suspensa's* components in abnormal splenic lymphocyte proliferation induced by ConA and LPS, we identified a beneficial component known as PHI with an IC50 of 4.359 and 1.539 μg/mL, respectively. Additionally, PHI inhibited cytokine secretion in LPS‐stimulated THP‐1 cells, which motivated us to explore its therapeutic effect on clinical inflammatory diseases using mouse models.

In this study, we selected DSS‐ and TNBS‐ induced UC model. DSS acts as a chemical toxin on the colon epithelium, resulting in epithelial damage and extremely similar to human UC, which is currently the most widely used colitis mouse model.^26^ TNBS is a kind of weak organic acid, which combined with intestinal mucosal keratin to form a whole antigen and stimulate intestinal immune response.^27^ Two animal models help more fully validate the therapeutic potential of PHI. As shown in Figures 2 and 3, significant therapeutic effects of PHI were found in mouse models of UC, as reflected by its ability to maintain body weight, reduce DAI and mortality, restore the intestinal mucosal barrier, and inhibit cytokine secretion. To investigate the protective effects of PHI on the gastrointestinal tract, H&E staining, PAS‐Alcian blue staining, and FITC‐dextran were used to prove the therapeutic effects of PHI on maintain intestinal morphology, reduce goblet cell loss, inflammatory cell infiltration, and decrease intestinal permeability. In IBDs, tight junction proteins play crucial roles by connecting epithelial cells, to maintain barrier function with mucus layer. In DSS‐induced colitis, the expression of tight junction‐associated protein was decreased, and PHI was able to restore the expression of E‐cadherin and Occludin as shown in Figure 2E, which reduced intestinal damage and restored mucosal barrier function. Furthermore, Macrophages are involved in regulating the progression of inflammation and the process of disease remission during intestinal repair. Flow cytometry analysis of MLN and PBMC showed that PHI could reduce macrophage infiltration in both colitis models. Immunofluorescence performed on colon tissues also demonstrated that PHI significantly inhibited macrophage infiltration in DSS‐induced colitis (Figure 4E). Cytokines are also important mediators in UC's enhancement and continuation, and their presence directly causes disease‐specific immune responses in UC, and we found that PHI could effectively inhibit the secreted level of proinflammatory cytokines in serum and colon, especially the secretion of IL‐1β.

The activation of IL‐1β in response to infection, mucosal injury, and stress triggers a local mucosal immune response, recruiting neutrophils to the affected site and activating the NF‐κB pathway, which leads to the upregulation of proinflammatory cytokines and chemokines.^5^ Furthermore, IL‐1β is the most likely effector molecule downstream of the NLRP3 inflammasome. Given the significant impact of PHI on macrophages and IL‐1β, we focused on the mechanism of action of the NLRP3 inflammasome and found that PHI inhibited inflammasome protein levels at both the genetic and protein levels. Starting with its upstream pathway, macrophages exposed to stimuli, such as ligands for TLR4, activate the transcription factor NF‐κB, which then upregulates NLRP3 expression.^28^ Specifically, TLR4 and MyD88 expression increased, and the phosphorylation of NF‐κB increased markedly, but PHI inhibited this pathway and restrained NLRP3 inflammasome activation. Finally, we conducted in vitro explorations with BMDM and found that PHI suppressed inflammation by inhibiting the activation of the NLRP3 inflammasome through the TLR4/MyD88/NF‐κB pathway.

## 5 CONCLUSION

In conclusion, PHI treatment exhibited significant improvements in UC reflected in reducing DAI, maintaining intestinal mucosal barrier, reducing inflammatory cell infiltration, and inhibiting the secretion of proinflammatory factors. The underlying mechanism is inhibited the activation of the NLRP3 inflammasome via the TLR4/MyD88/NF‐κB pathway. These findings provide potential mechanisms underlying the therapeutic effects of PHI on UC.

## AUTHOR CONTRIBUTIONS

Xiao Tong and Li Chen performed most of the experiments. Shuangshuang Liu, Jiaying Yao, and Zhenguang Shao performed some experiments. Shijun He, Sheng Yao, Yang Ye, Zemin Lin, and Jianping Zuo designed the experiments. Shijun He and Jianping Zuo interpreted the results. Xiao Tong and Zemin Lin wrote the paper.

## CONFLICT OF INTEREST STATEMENT

The authors declare no conflict of interest.

## ETHICS STATEMENT

All procedures followed the National Institutes of Health Guide for Care and Use of Laboratory Animals and were approved by the Bioethics Committee of the Shanghai Institute of Materia Medica (IACUC Protocol #2021‐04‐ZJP‐145; #2021‐07‐ZJP‐151; #2022‐01‐ZJP‐161; #2022‐06‐ZJP‐169; #2022‐06‐ZJP‐177). We declare that the publisher has the author's permission to publish the relevant contribution.

## Supporting information

Supporting information.

## Data Availability

The data sets analyzed during the current study are available from the corresponding author on reasonable request.
