# Supplementary material for: Forsythia suspensa (Thunb.) Vahl extract ameliorates ulcerative colitis via inhibiting NLRP3 inflammasome activation through the TLR4/MyD88/NF‐κB pathway
Source: Immun Inflamm Dis. 2023 Nov 7;11(11):e1069. doi: 10.1002/iid3.1069 (PMC10629261; doi:10.1002/iid3.1069)
Supplement: Supplementary file 1 — Supporting information. [file IID3-11-e1069-s001.docx]

**Supplementary Materials**

**Fig. S1: The purity of naturally occurring phillygenin.**


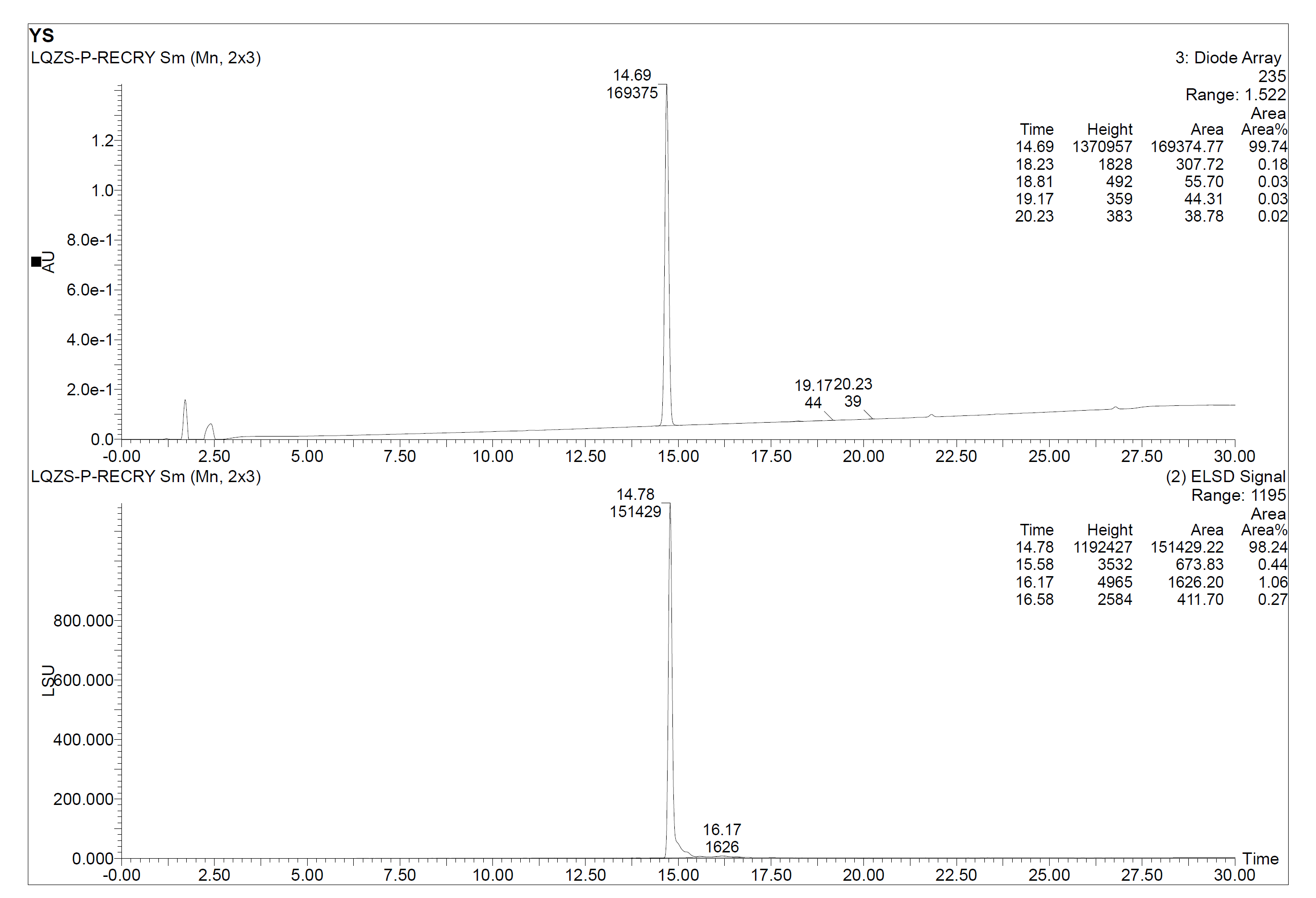


**Fig. S2: The purity of synthetic phillygenin.**


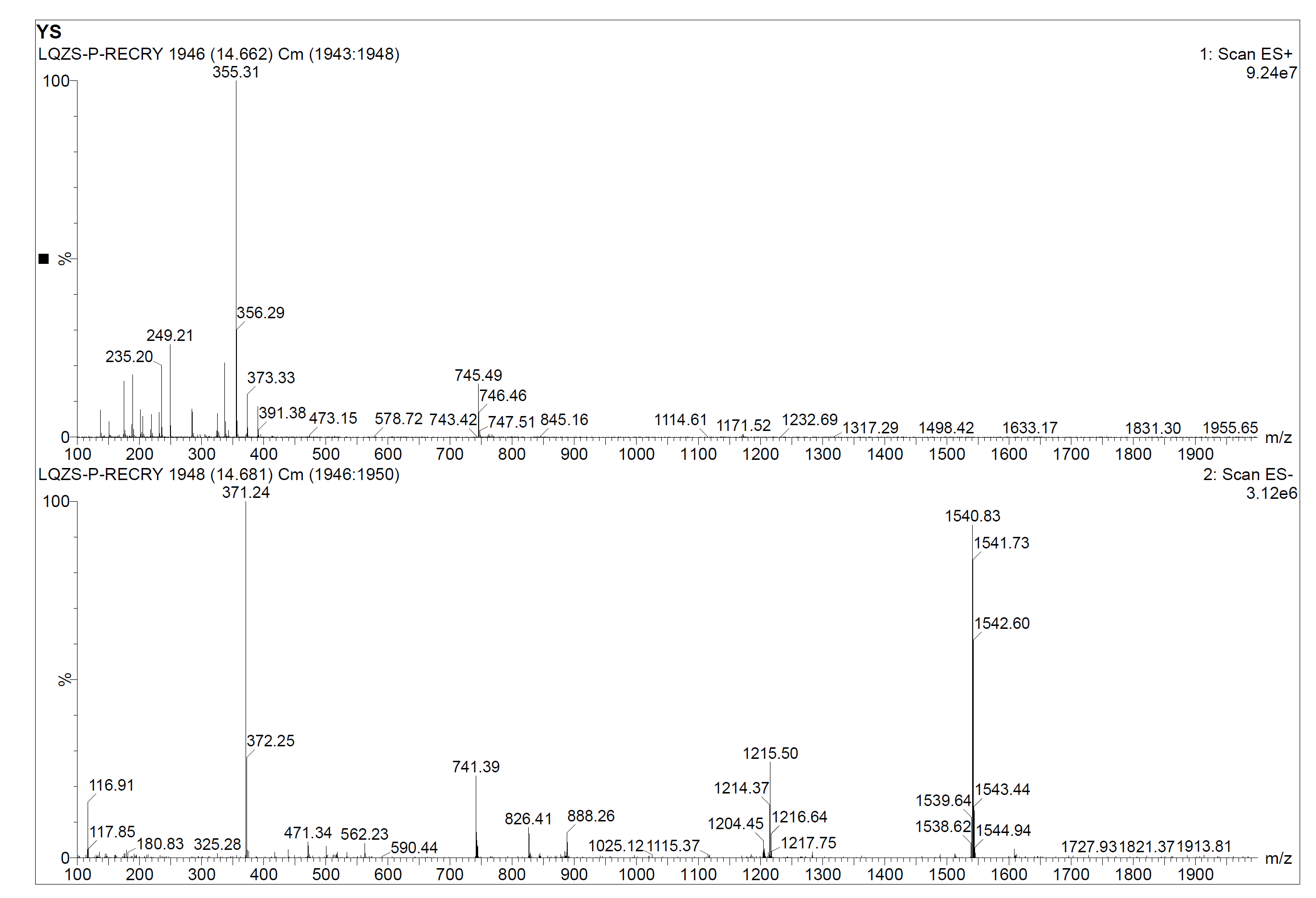


**Fig. S3: ESI-MS of phillygenin.**

**Fig. S4: ^1^H NMR (500 MHz, CDCl_3_) of phillygenin.**

**Fig. S5: ^1^H NMR (125 MHz, CDCl_3_) of phillygenin.**
